# Interstitial cell network volume is reduced in the terminal bowel of ageing mice

**DOI:** 10.1111/jcmm.13794

**Published:** 2018-07-25

**Authors:** Prasanna P. K. M. Gamage, Bhavik A. Patel, Mark S. Yeoman, Rachel N. Ranson, M. Jill Saffrey

**Affiliations:** ^1^ School of Life, Health and Chemical Sciences Open University Milton Keynes UK; ^2^ School of Pharmacy and Biomolecular Sciences University of Brighton Brighton UK; ^3^ Centre for Stress and Age‐Related Disease University of Brighton Brighton UK; ^4^ Department of Applied Sciences Faculty of Health and Life Sciences Northumbria University Newcastle upon Tyne UK; ^5^ Present address: Biomedical Engineering Program Department of Electronic & Telecom. Engineering Faculty of Engineering General Sir John Kotelawala Defence University Ratmalana Sri Lanka

**Keywords:** autonomic nervous system, constipation, enteric nervous system, gastrointestinal tract, IAS, incontinence, internal anal sphincter, smooth muscle

## Abstract

Ageing is associated with impaired neuromuscular function of the terminal gastrointestinal (GI) tract, which can result in chronic constipation, faecal impaction and incontinence. Interstitial cells of cajal (ICC) play an important role in regulation of intestinal smooth muscle contraction. However, changes in ICC volume with age in the terminal GI tract (the anal canal including the anal sphincter region and rectum) have not been studied. Here, the distribution, morphology and network volume of ICC in the terminal GI tract of 3‐ to 4‐month‐old and 26‐ to 28‐month‐old C57BL/6 mice were investigated. ICC were identified by immunofluorescence labelling of wholemount preparations with an antibody against c‐Kit. ICC network volume was measured by software‐based 3D volume rendering of confocal Z stacks. A significant reduction in ICC network volume per unit volume of muscle was measured in aged animals. No age‐associated change in ICC morphology was detected. The thickness of the circular muscle layer of the anal sphincter region and rectum increased with age, while that in the distal colon decreased. These results suggest that ageing is associated with a reduction in the network volume of ICC in the terminal GI tract, which may influence the normal function of these regions.

## 1 INTRODUCTION

Impaired function of the terminal bowel is common in the elderly and can result in chronic constipation, faecal impaction and incontinence, reducing quality of life.^1^ Previous studies have described changes in the innervation and smooth muscle of the ageing gut,^2^ but analysis of changes in other cell types involved in regulation of smooth muscle motility in the terminal bowel (ie the anal sphincter region, or ASR, rectum and distal colon) is limited. In the ageing mouse, impaired colonic motility,^3^ faecal output^4^ and reduced frequency and amplitude of smooth muscle contraction in the anorectum [MS in Press] have been measured. The cellular changes underlying these functional impairments, however, remain to be fully determined.

As in other parts of the gut, movement of contents along the terminal bowel occurs as a result of co‐ordinated activity of intestinal smooth muscle, which is regulated by the enteric nervous system and by two specialised types of stromal cells: interstitial cells of cajal (ICC)^5^ and PDGFRα+ve (platelet‐derived growth factor receptor alpha‐positive cells), also referred to as Telocytes, or fibroblast‐like cells.^6^ Defaecation involves involuntary relaxation of the smooth muscle of the internal anal sphincter (IAS, a thickened region of circular muscle in the anal canal), which is also controlled by these three cell types.

Changes in the distribution and the morphology of ICC and PDGFRα+ cells during ageing have been little studied. In humans, a loss of ICC and a reduction in ICC network volume in normal stomach and colon with age have been described,^7^ while in ageing rats, a reduction in density of ICC has been reported in the proximal colon^8^ and stomach.^9^ A reduction in c‐Kit protein levels has also been described in the ageing rat colon.^10^ No studies to investigate the effects of age on the network volume of ICC in the terminal bowel or in the mouse gut have been performed. The aim of this study was therefore to determine whether changes in the network volume of ICC populations in the mouse terminal gut occur during ageing.

## 2 METHODS

Details of the materials and methods are provided in Data S1 and Figure S1. Five 3‐ to 4‐month‐old and five 26‐ to 28‐month‐old C57BL/6 male mice were used. All procedures were carried out according to United Kingdom Home Office regulations. Large intestine from the colo‐caecal junction to the anal orifice was excised and its length was measured. Smooth muscle relaxation was standardised using the calcium channel blocker nicardipine hydrochloride (10^−6^ mol/L; Sigma, Dorset, UK; N7510) for 15 minutes. Wholemounts were prepared and fixed for 30 minutes in ice‐cold acetone, then washed three times in PBS. Muscularis externa was separated from the other layers before immunolabelling by incubation in anti‐c‐Kit antibody (14‐1172; Life Technologies Ltd (Thermo Fisher Scientific‐UK), at Life Technologies Ltd, Paisley, Scotland UK; 2 μg/mL) overnight at room temperature. Samples were then washed in PBS 4× 30 minutes, incubated in secondary antibody, goat anti‐rat Alexa 555 (6 μg/mL; Life Technologies Ltd (Thermo Fisher Scientific‐UK), at Life Technologies Ltd, Paisley, Scotland UK; 21434) in darkness at room temperature for 2 hours. Quantification of c‐Kit immunopositive ICC volume was performed using methods and software based on previous studies.^7^, ^11^ Results were corrected for changes in gut size with age. ICC network volumes per unit volume of tissue were expressed as mean ± SEM. Comparison of different age groups using unpaired two tailed *t* test was performed in GraphPad Prism software (La Jolla, CA, USA). *P* values <0.05 were considered statistically significant.

## 3 RESULTS

### 3.1 Distribution and morphology of ICC in the terminal bowel

**Figure 1:**
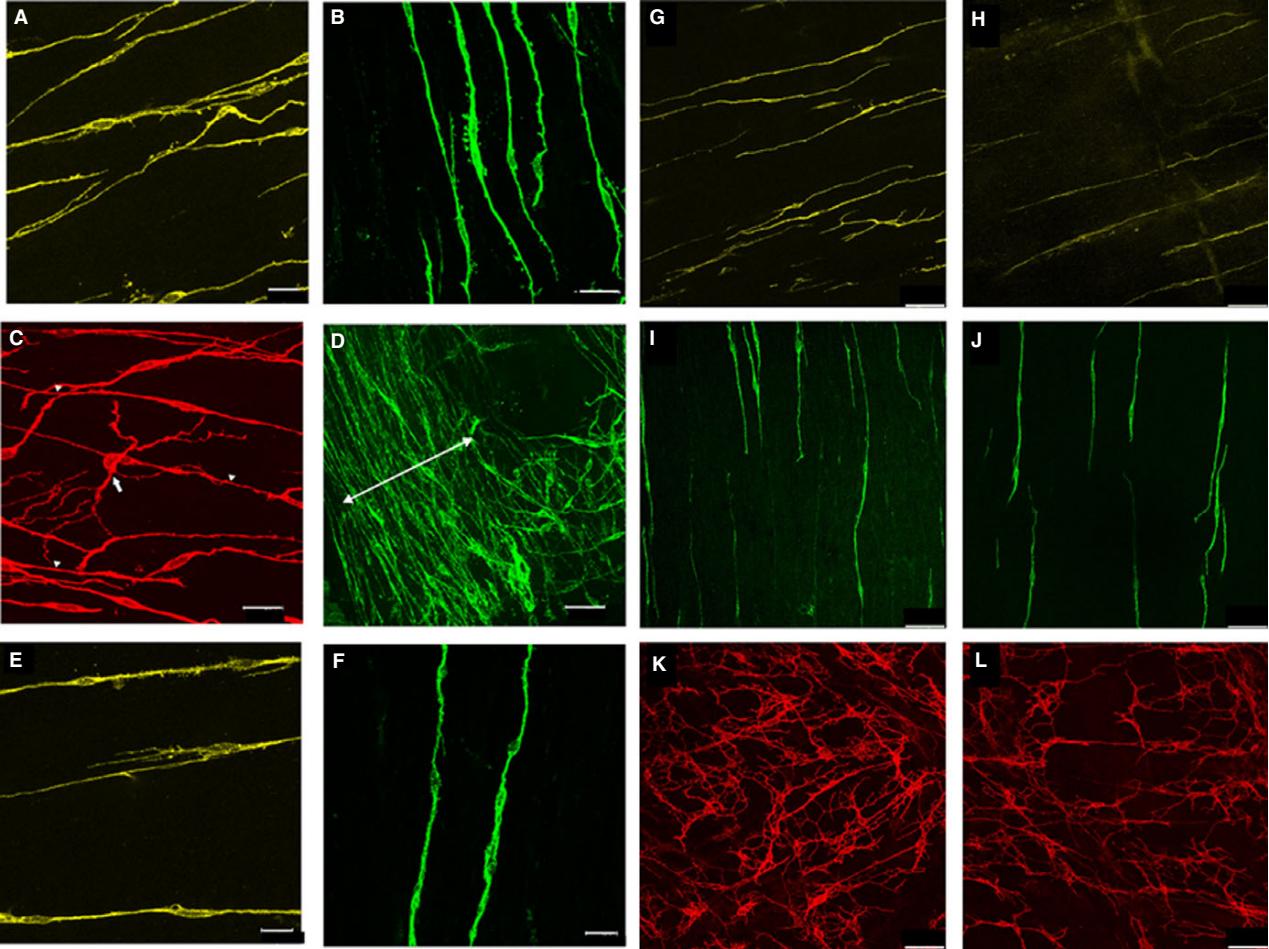
A‐F, Morphology of ICC in the ASR of 3‐ to 4‐mo (A‐D) and 26‐ to 28 (E,F)‐month‐old mice. (A,E) ICC‐LM. (B,F) ICC‐CM (C) An isolated ICC‐MY multipolar cell (arrow) is visible 200 μm from the anal verge. ICC‐LM are also visible in this image (arrowheads). (D), Densely populated band (double headed arrow) of ICC‐CM at the most aboral end of the IAS. G‐L, Comparison of the ICC in the rectum of 3‐ to 4‐mo (G,I,K) and 26‐to 28 (H,J,L)‐month‐old mice. (G,H) ICC LM, (I,J) ICC CM, (K,L) ICC MY

In the ASR, c‐Kit immunoreactive cells were observed in both the longitudinal and circular muscles (Figure 1A,B). These cells were bipolar with an elongated spindle shape and were oriented parallel to the long axis of smooth muscle cells. A densely populated band of ICC‐CM (Figure 1D) was observed at the most aboral end of the ASR. This area was small, variable in size and not homogeneous, so not suitable for quantification. Oral to this band, the distribution of ICC was homogenous, but less dense. Fine processes of ICC‐CM in the ASR appeared to have close contacts with the processes of other ICC‐CM in a linear arrangement along the axis of smooth muscle cells (Figure 1B). ICC‐MY were very rarely seen, at the interface between the two muscle layers (Figure 1C). Therefore, a network of ICC‐MY was not found in the mouse ASR.

In the rectum and distal colon, c‐Kit immunoreactive ICC‐LM, ICC‐CM and ICC‐MY were observed (Figures 1G,I,K and S1). The shape of ICC‐LM was similar to those in the ASR and primary processes of some branched into shorter secondary processes (eg Figure 1G). Fusiform, bipolar ICC‐CM and oriented parallel to the long axis of circular muscle cells were observed throughout the circular muscle layer (Figure 1I,J). Like ASR ICC‐CM, rectal ICC‐CM appeared to form linear connections with the processes from adjacent ICC‐CM. ICC‐CM in the distal colon, however, were comparatively shorter, broader and had small spine‐like protrusions in their long primary processes (Figure S1B). ICC‐MY in the rectum were scantly distributed at the aboral end, adjacent to the ASR, but appeared to gradually increase in number in the aboral to oral direction. Pilot studies showed that there were very few or no ICC‐MYs until approximately 900 μm from the anal verge. Hence, at the oral end of the rectum, a dense and a complex network of ICC‐MY, with overlapping processes from adjacent cells, was observed (Figure 1K). Multipolar ICC‐MY usually had three to five primary cytoplasmic processes that branched further into secondary and tertiary processes, which were very close to processes of adjacent cells (Figure 1K).

### 3.2 Effect of age on the morphology and network volume of c‐Kit labelled cells

In 26‐ to 28‐month‐old animals, the overall appearance of ICC in the ASR (Figure 1E,F), rectum (Figures 1H,J,L) and distal colon (Figure S1) was similar to those in 3‐ to 4‐month‐old animals. Although there appear to be more “spaces” in the network of ICC‐MY of old animals (Figures 1L and S1F), it is important to note that these images were obtained in tissue preparations that had undergone differing degrees of stretch. Hence, it is not possible to compare the network volume of ICC populations between different samples visually.

**Figure 2:**
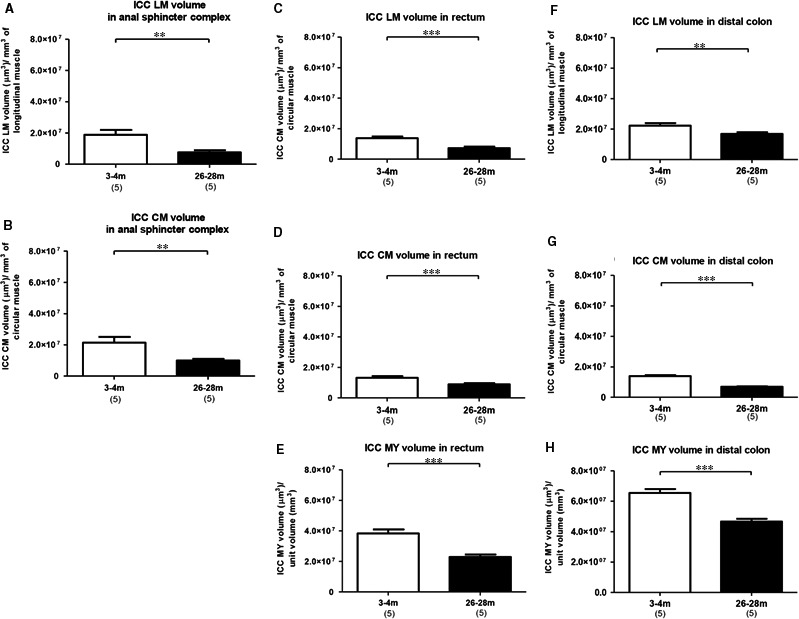
Change in ICC network volume (μm^3^) per unit volume (mm^3^) of muscle with age in 3‐ to 4‐mo and 26‐ to 28‐mo‐old animals. (A, B): ASR. (C‐E): Rectum, (F‐H): Distal colon. The number of animals used in each age group is shown in brackets. Mean + SEM values are shown. *P* values from unpaired two tailed *t* test, ** *P* < 0.01; *** *P* < 0.001

To measure ICC network volume, the thicknesses of the circular and longitudinal muscle layers were determined using data from confocal Z stacks (Figure S2). In the ASR, the c‐Kit immunoreactive ICC‐LM and ICC‐CM network volumes (μm^3^) per unit volume (mm^3^) of muscle showed a significant reduction (*P* < 0.01) in 26‐ to 28‐month‐old compared with 3‐ to 4‐month‐old animals (Figure 2A,B). As ICC‐MY in the ASR were only infrequently seen, statistical comparison of ICC‐MY volumes in the ASR between the two age groups was not possible. The ICC‐LM, ICC‐CM and ICC‐MY network volumes in the rectum and distal colon in 26‐ to 28‐month‐old animals were significantly smaller (*P* < 0.001) than in 3‐ to 4‐month‐old animals (Figure 2C‐H).

## 4 DISCUSSION

The present study is the first to investigate the effects of age on ICC populations in the mouse terminal bowel. A significant reduction in ICC network volume was measured in old animals, in all three regions. The distribution of ICC observed in the mouse terminal bowel was in keeping with that described previously, apart from the observation of a small area of densely arranged ICC‐CM in the most distal part of the ASR, which has not previously been described. This region is of interest because the frequency and amplitude of slow waves has been reported to be greatest in the most distal region of the IAS.^12^ In the mouse IAS, ICC‐MY have been reported to be few in number, not form a network and to decline distally.^12^, ^13^, ^14^ In keeping with these previous studies, only a very small number of ICC‐MYs were observed in the ASR. These ICC‐MY did not form a network, and their number was too small to allow meaningful quantification.

Changes in ICC volume in smooth muscle could be because of a reduction in the expression of cKit by ICC during ageing, and/or a reduction in ICC numbers and/or individual ICC volume. It could also be as a result of failure of ICCs to increase in volume in proportion to increases in muscle volume. In this study (Data S1), no changes in LM volume with age were detected, but the CM volume in the ASR and rectum increased, while that in the distal colon decreased in old mice. Thus, the reduction in ICC‐LM network volume in old mice is likely to be because of changes in the ICC population, while changes in the ICC‐CM volume in the ASR and rectum may be the result of change in CM layer thickness as well as ICC changes. In the case of the distal colon CM, in which both muscle thickness and ICC‐CM volume were reduced, age‐related changes in the ICC population were most marked.

An age‐associated reduction in ICC density has been reported in human colon and stomach,^7^ and rat proximal colon^8^ and stomach.^9^ The results obtained here for the mouse terminal bowel are therefore in line with previous reports on other parts of the GI tract in two other species, indicating that a reduction in ICC network volume during ageing may be a general phenomenon. Evidence that ICC play a critical role in regulation of smooth muscle contractility is now widely accepted^15^ including in the terminal bowel.^13^ These results therefore suggest that changes in ICCs during ageing described here may be an important factor in the physiological changes observed in this region.

## CONFLICT OF INTEREST

The authors confirm that there are no conflict of interests.
