# Supplementary figures and images for: Interstitial cell network volume is reduced in the terminal bowel of ageing mice
Source: J Cell Mol Med. 2018 Jul 25;22(10):5160–4. doi: 10.1111/jcmm.13794 (PMC6156346; doi:10.1111/jcmm.13794)

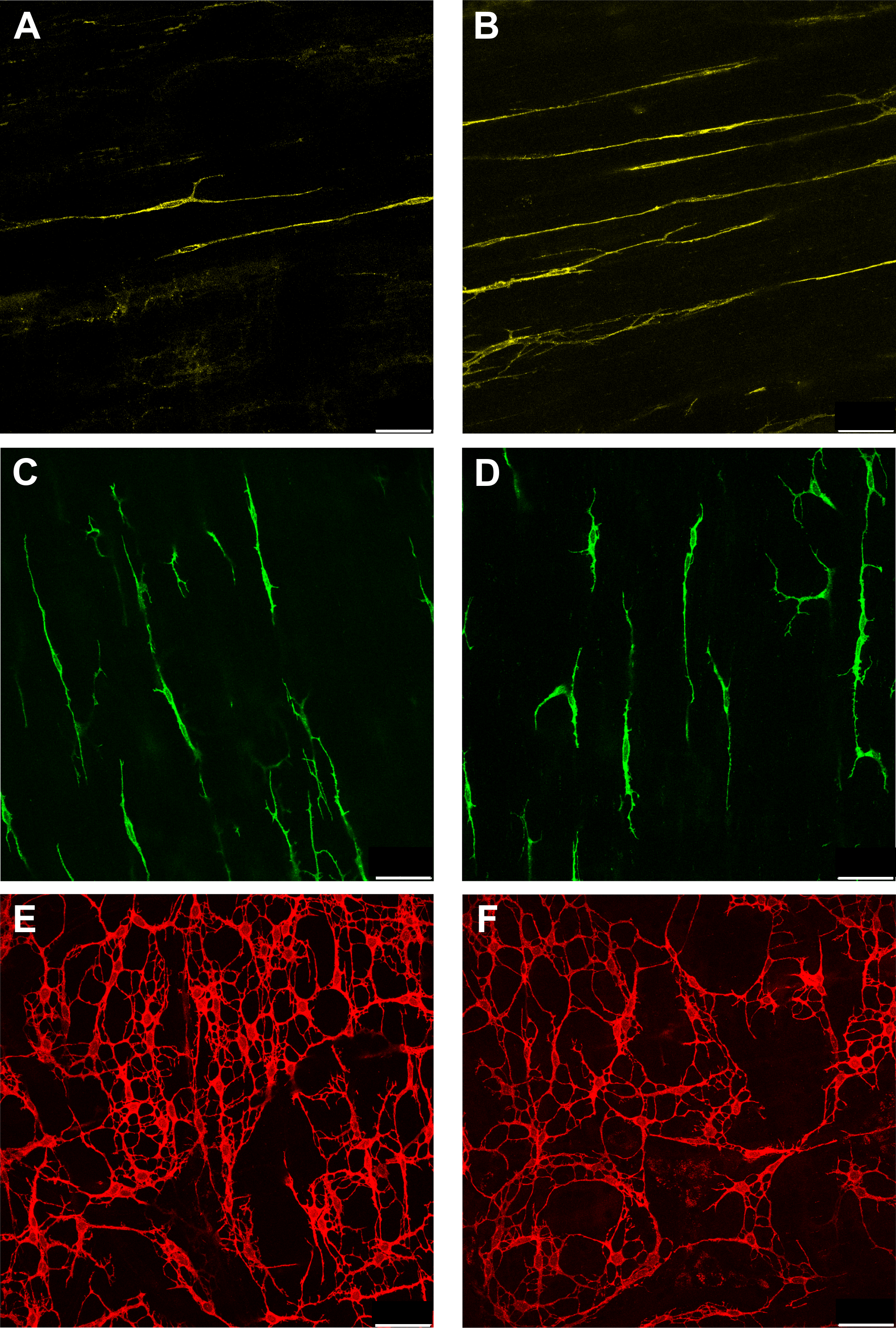

Supplement: Supplementary file 1 [file JCMM-22-5160-s001.tif]

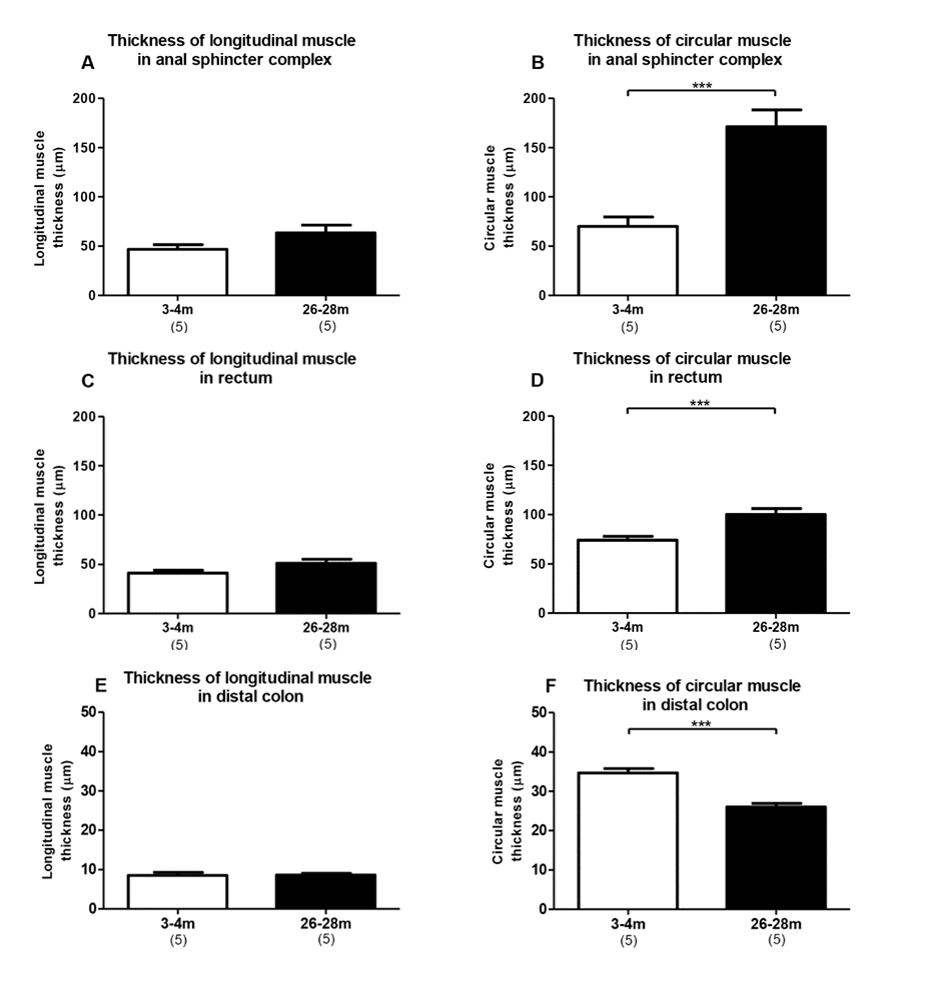

Supplement: Supplementary file 2 [file JCMM-22-5160-s002.tif]
