# Supplementary material for: Interstitial cell network volume is reduced in the terminal bowel of ageing mice
Source: J Cell Mol Med. 2018 Jul 25;22(10):5160–4. doi: 10.1111/jcmm.13794 (PMC6156346; doi:10.1111/jcmm.13794)
Supplement: Supplementary file 3 [file JCMM-22-5160-s003.docx]

**Supplementary Material 1: Materials and Methods**

**1.1 Animals**

C57BL/6 male mice were obtained from Harlan, UK at 8 weeks of age. Mice were housed in groups of 5 and maintained at 19-23^0^C, 50% +/-10% humidity on a 12 hour light and 12 hour dark cycle. RM1 (E) 801002 (Special Diet Services) chow and UV sterilized mains water were provided *ad libitum.* Five 3-4 month and five 26-28 month old mice were used in the study. All procedures were carried out according to U.K. Home Office regulations and were approved by the Open University Ethics Committee.

**1.2 Sample collection and preparation**

Animals were weighed then killed by CO_2_ (100%) asphyxiation. The abdomen was opened along the midline, the large intestine from the colo-caecal junction to the anal orifice was excised and its length was then measured in a tray filled with phosphate buffered saline (PBS; 0.85% NaCl in 0.01 mol L^-1^ sodium phosphate buffer, pH 7.4), allowing it to float freely. The proximal 70% of C57BL/6 mouse large intestine was considered colon (comprising 50% proximal and 50% distal colon) and the remaining 30% of the large intestine was considered the ano-rectum including the ASR. The total average length of the distal colon in 3-4 month old C57BL/6 mice was at least 2.41 cm. Therefore, to take a representative sample of the distal colon of mouse gastrointestinal tract, an approximately 2 cm segment aboral to the midpoint of the large intestine was taken. The total average length of the ano-rectum in young adult (3-4 month old) C57BL/6 mice was at least 2.0 cm from the anal verge. Therefore, to sample ano-rectum including ASR, an approximately 0.6 cm segment oral to the anal verge was taken.

A representative region of the ASR, up to 1mm from the anal verge in 3-4 m and up to 1.5 mm from the anal verge in 26-28 m samples, was taken. The region between the oral end of the ASR up to 4.1mm from the anal verge in 3-4 m and up to 4.6 mm from the anal verge in 26-28 m animals was taken as the representative region of the rectum. For the distal colon, a length of 9 mm from the midpoint of the large intestine in 3-4 month old animals was taken as the representative region. In 26-28 month old animals however, the length considered from the midpoint of the large intestine varied according to the age-specific length correction factor [= 9 x Age-specific length correction factor].

Excised samples were processed as previously described [Gamage et al., *Neurogastroenterol Motil.* 2013; 25(7):e495-e505. DOI: 10.1111/nmo.12114]. To ensure maximal smooth muscle relaxation and hence standardize smooth muscle relaxation between preparations, samples were placed in PBS containing the calcium channel blocker nicardipine hydrochloride (10^-6^ mol L^-1^; Sigma, Dorset, England; N7510) for 15 minutes. Luminal contents were carefully flushed out using a syringe filled with PBS. The specimens were then opened by cutting longitudinally along the mesenteric attachment and laid on laminated graph paper to measure their circumference (C, mean of measurements taken at each end of the specimen) and length (L). Specimens were then separated into two parts (wholemount ‘I’ and ‘II’) by cutting longitudinally along the anti-mesenteric line, and wholemount I was used for the investigation of ICC in this study (wholemount II was used for other work). It was then maximally stretched and pinned mucosal side down on Sylgard (Ellsworth Adhesives, Glasgow; Sylgard184) -lined plates and immersion fixed for 30 minutes in ice cold Acetone (BDH Lab Supplies, Broom Road, Poole, England; 27023) on ice, followed by 3 x 10 minutes washes in PBS. The width of the tissue preparations (C’) and the length (L’) following fixation and washing were also measured as above. To prepare wholemounts for immunolabelling, fixed tissues were pinned to Sylgard-lined plates with the mucosal side down, and under the dissecting microscope, the muscularis externa was separated from the other layers.

**1.3 c-Kit immunolabelling**

Wholemount preparations were first incubated in antibody diluting solution (ABDS; lysine 1mg/ml, 0.01% bovine serum albumin and 0.05% sodium azide in PBS. pH 7.4) containing 0.5% Triton X-100 (Sigma-Aldrich, UK; X100) to aid antibody penetration, for 1 hour at room temperature. This was followed by incubation in 10% normal goat serum (Dako, UK; X0907) in ABDS with 0.1% Triton X 100 for 2 hours at room temperature to reduce non-specific binding of antibodies. Wholemounts were then incubated in anti c-Kit antibody (14-1172; eBioscience; 2μg/ml) in ABDS with 0.1% Triton X 100 overnight (18 hours) at room temperature. After incubation in primary antiserum, wholemounts were washed in PBS 4 x 30 minutes each, before incubating in secondary antibody, goat anti rat Alexa 555 (6μg/ml; Invitrogen; 21434) in darkness at room temperature for 2 hours. Wholemounts were then washed in PBS (4 x 30 minutes) and mounted on glass slides (Superfrost Plus, Thermo Scientific), cover-slipped with Citifluor (Agar Scientific Limited, Stansted, UK; R1320) mounting medium and the edges of cover slips were sealed using clear sealant.

*Determination of optimal labelling conditions and control experiments*

Pilot experiments to determine the optimum dilutions of antisera were performed for the primary antiserum. Control experiments in which anti c-Kit primary antibodies were omitted produced no unspecific labelling. When inappropriate secondary antisera were applied, no labelling was observed.

**1.4 Confocal Microscopy**

The methods and software used for the quantitative analysis performed in this study were based on those used in similar previous studies [See References 7 and 11 in main text and Gamage et al, 2013, above]. Immunolabelled wholemount preparations were examined by an experimenter blind to the age of specimens. Fluorescent images of Alexa 555 binding were acquired by using a 605/ 632-nm emission filter. To obtain quantitative data on smooth muscle thickness and ICC density, confocal Z stacks (1024 × 1024 pixels; optical section 1.51 μm) spanning the entire thickness of the muscularis externa were captured at regular intervals in transverse strips across the entire samples using a confocal laser scanning microscope (Leica DM6000 CS) equipped with a x40 objective, which defined an area of (387.5 x 387.5 μm^2^) on the specimen. Midpoints of two adjacent strips on stretched tissue were 2 mm apart. Within a sampling strip the whole width of the strip from mesenteric attachment to anti -mesenteric aspects was sampled with at most 0.775 mm gap between two adjacent Z stacks. For the ano-rectum, seven transverse strips starting from the most aboral end of the wholemount were sampled. For the distal colon, 10 transverse strips starting from the most oral end of the wholemount were sampled. Quantitative and qualitative analysis of confocal Z stacks were also performed by an experimenter blind to the age of the tissue from which the Z stacks were taken.

**1.5 Measurement of smooth muscle thickness and quantification of c-Kit immunopositive ICC volume**

*Calculation of smooth muscle thickness:*

The thickness of the muscle layers was determined from the number of 1.51μm Z stacks required to sample throughout each muscle layer. The volume occupied by c-Kit-positive ICC networks was calculated from the confocal stacks using ANALYZE software (Mayo Foundation, Rochester, MN, USA). Each confocal Z stack was 3D volume rendered and thresholded to obtain ICC volumes for ASR, rectum and distal colon separately.

*Correction for change of size of the gut with age*

The lengths of the ASR, rectum and distal colon vary with the age of the animals. Thus, an age-specific length correction factor [mean length of the large intestine of 26-28 month old animals/ mean length of the large intestine of 3-4 month old animals] was employed to calculate the size of ASR, rectum and distal colon for each of the animals in the two age groups. As the degree of stretch of wholemounts varies, even of samples from animals of the same age, the actual distance between midpoints of transverse strips varies. Thus a stretch-specific length correction factor was employed [length of wholemount after nicardipine incubation and before stretching/ length of wholemount after fixation and stretching], and the actual distance between midpoints of transverse strips in each of the wholemounts were determined.

*Correction for stretch of wholemounts*

Wholemounts from animals of the same age group were stretched to varied extents. Hence, the area measurements of wholemounts were corrected using a stretch correction factor [C x L]/ [C’ x L’]. The stretched volume of muscle for each of the Z stacks was divided by the un-stretched area [A= 0.150 x stretch correction factor mm^2^] of the respective Z stack, to obtain the un-stretched thickness of muscular layers.

*Calculation of ICC volume*

The volume of each Z stack could be determined from its measured parameters. The stretched thickness of the longitudinal and circular muscles [Number of Z steps x Z step size] (muscularis externa) and the volume of muscle [Number of Z steps x Z step size x 0.150 mm^2^] for each of the Z stacks was calculated. Therefore it was possible to calculate the ICC volume per unit volume of muscle [ICC volume/ muscle volume of the respective Z stack] for each of the Z stacks. Values thus obtained for each region in 3-4m animals were statistically compared with those from 26-28m animals.

**1.6 Statistical analysis**

ICC network volumes per unit volume of tissue were expressed as mean ±SEM. Comparison of different age groups using unpaired two tailed t test was performed in GraphPad prism software (Avenida de la Playa La Jolla, USA). P values <0.05 were considered statistically significant.

**1.7 Application of false colours to images**

All the images obtained in the confocal laser-scanning microscope had a fluorescence signal in the red channel. Hence, all the labeled ICC in a particular confocal Z stack appeared in red. In order to facilitate the easy identification of ICC-CM, -MY and -LM populations, a false colour palette was applied to ICC-LM (yellow) and ICC-CM (green) populations. This however, was not possible in one area, the very terminal part of the ASR (Figure 1C), where the boundaries of three ICC populations could not be clearly be demarcated in a stack.
