# Supplementary material for: Interstitial cell network volume is reduced in the terminal bowel of ageing mice
Source: J Cell Mol Med. 2018 Jul 25;22(10):5160–4. doi: 10.1111/jcmm.13794 (PMC6156346; doi:10.1111/jcmm.13794)
Supplement: Supplementary file 4 [file JCMM-22-5160-s004.docx]

**Supplementary Figure Legends:**

**Supplementary Figure 1**: Comparison of the ICC in the distal colon of 3-4 (A, C, E) and 26-28 (B, D, F) month old mice. (A, B) ICC LM, (C, D) ICC CM, (E, F) ICC MY

**Supplementary Figure 2:** Mean thickness (µm) of muscle layers in the ASR, rectum and distal colon of mice aged 3-4 and 26-28 months. (A) Longitudinal muscle in the ASR. (B) Circular muscle in the ASR. (C) Longitudinal muscle in the rectum. (D) Circular muscle in the rectum. (E) Longitudinal muscle in the distal colon. (F) Circular muscle in the distal colon. The number of animals used in each age group is shown in brackets. Mean + SEM values are shown. p values from unpaired two tailed t test, *** indicates p< 0.001.
